# Supplementary material for: NPM1 upregulates the transcription of PD-L1 and suppresses T cell activity in triple-negative breast cancer
Source: Nat Commun. 2020 Apr 3;11:1669. doi: 10.1038/s41467-020-15364-z (PMC7125142; doi:10.1038/s41467-020-15364-z)
Supplement: Supplementary file 1 — Supplementary Information [file 41467_2020_15364_MOESM1_ESM.pdf]

NPM1 upregulates the transcription of PD-L1 and suppresses T-cell activity in triple negative breast cancer

Ge Qin et al.

Supplementary table 1: Correlation analysis of PD-L1 expression and clinical features in 149 breast cancer patients.

	PD-L1 expression 〔n(%)〕		
Variable	Negative	Positive	P-value
Median age (years)	47 (27~78)		
	46 (27~75)	47.5(30~78)	
Tumor size			0.001*
≤ 20 mm	39 (84.8)	7 (15.2)	
＞20 mm	60 (58.3)	43 (41.7)	
Grade			0.191
I	24 (77.4)	7 (22.6)	
II~III	62 (63.3)	36 (36.7)	
LN metastasis			0.863
Negative	50 (67.6)	24 (32.4)	
Positive	49 (65.3)	26 (34.7)	
Positive lymph			0.613
nodes	54 (69.2)	24 (30.8)	
0	17 (56.7)	13 (43.3)	
1~3	15 (71.4)	6 (28.6)	
4~9	13 (65.0)	7 (35.0)	
≥10			
Distant metastasis			0.078
Negative	89 (69.5)	39 (30.5)	
Positive	10 (47.6)	11 (52.4)	
Receptor status			＜0.001**
ER			
Negative	24 (42.1)	33 (57.9)	
Positive	75 (81.5)	17 (18.5)	
PR			
Negative	21 (39.6)	32 (60.4)	＜0.001**
Positive	78 (81.2)	17 (18.5)	
HER-2			0.165
Negative	90 (64.7)	49 (35.3)	
Positive	9 (90.0)	1 (10)	
Ki67 index			0.726
≤14%	40 (64.5)	22 (35.5)	
＞14%	59 (67.8)	28 (32.2)	
Subtypes			＜0.001*
Non-TNBC	79 (81.4)	18 (18.6)	
TNBC	20 (38.5)	32 (61.5)	

*Positive correlation

**Negative correlation

Data was analyzed by two-side Pearson Chi-square test.

Supplementary table 2: Univariate and multivariate analysis for OS.

Variables	Univariate				Multivariate			
		95% CI				95% CI		
	HR	Lower	Upper	P-value	HR	Lower	Upper	P-value
Age (continue)	0.989	0.973	1.006	0.196				
Tumor size (＞20 vs.≤20 mm)	1.458	1.000	2.127	0.046	1.035	0.581	1.845	0.907
Grade (II~III vs. I)	1.669	1.092	2.551	0.014	1.099	0.553	2.183	0.787
LN metastasis (pos vs. neg)	1.481	1.028	2.133	0.037	1.440	0.881	2.355	0.146
Distant metastasis (pos vs. neg)	3.383	1.477	7.746	0.011	1.906	0.721	5.040	0.194
ER status (pos vs. neg)	0.561	0.387	0.814	0.003	0.215	0.050	0.913	0.037
PR status (pos vs. neg)	0.607	0.416	0.886	0.012	0.185	0.022	1.526	0.117
HER-2 status (pos vs. neg)	1.906	0.991	3.666	0.076				
Ki67 index（＞14% vs. ≤14%）	2.220	1.475	3.343	0.000	2.157	1.377	3.380	0.001
PD-L1 status (pos vs. neg)	1.266	0.850	1.884	0.254				
TNBC (yes vs. no)	1.619	1.107	2.368	0.016	0.070	0.005	0.945	0.045

Data was analyzed by Likelihood Ratio (LR) test.

Supplementary table 3: Univariate and multivariate analysis for OS.

Variables	Univariate				Multivariate			
	HR	95% CI Lower	95% CI Upper	P-value	HR	95% CI Lower	95% CI Upper	P-value
Age (continue)	1.060	1.026	1.096	<0.001	1.079	1.043	1.117	<0.001
Grade (II~III vs. I)	2.895	0.872	9.618	0.083				
LN metastasis (pos vs. neg)	2.418	1.086	5.383	0.031	2.526	0.997	6.398	0.051
Distant metastasis (pos vs. neg)	11.236	4.726	26.714	<0.001	9.876	3.854	25.308	<0.001
ER status (pos vs. neg)	0.261	0.117	0.582	0.001	0.224	0.074	0.681	0.008
PR status (pos vs. neg)	0.407	0.140	1.185	0.099				
HER-2 status (pos vs. neg)	1.200	0.484	2.973	0.694				
NPM1 status (low vs. high)	2.361	1.105	5.047	0.027	1.991	0.801	4.954	0.138
PD-L1 status (pos vs. neg)	2.815	1.264	6.266	0.011	1.611	0.575	4.508	0.364
TNBC (yes vs. no)	3.086	1.444	6.596	0.004	1.033	0.304	3.507	0.958

Data was analyzed by Likelihood Ratio (LR) test.

Supplementary table 4: Correlation analysis of NPM1 expression and clinical features in 133 breast cancer patients.

Variable	NPM1 expression 〔n(%)〕 Low	High	P-value
Median age (years)	54 (29~87)		
	53 (29~87)	55 (34~86)	
Grade			0.726
I	20 (60.6)	13 (39.4)	
II~III	64 (64.0)	36 (36.0)	
LN metastasis			0.315
Negative	38 (58.5)	27 (41.5)	
Positive	39 (67.2)	19 (32.8)	
Positive lymph			0.195
nodes	38 (58.5)	27 (41.5)	
0	21 (75.0)	7 (25.0)	
1~3	16 (66.7)	6 (33.3)	
4~9	2 (33.3)	4 (66.7)	
≥10			
Distant metastasis			0.205
Negative	65 (66.3)	33 (33.7)	
Positive	19 (54.3)	16 (45.7)	
Receptor status			0.065
ER			
Negative	26 (53.1)	23 (46.9)	
Positive	58 (69.0)	26 (31.0)	
PR			
Negative	36 (54.5)	30 (45.5)	0.041**
Positive	48 (71.6)	19 (28.4)	
HER-2			0.578
Negative	67 (62.0)	41 (38.0)	
Positive	17 (68.0)	8 (32.0)	

*Positive correlation

**Negative correlation

Data was analyzed by two-side Pearson Chi-square test.

Supplementary table 5: Primer Information

Primers for PD-L1 promoter probe	Forward 5'-GGCTGCGGAAGCCTATTCTA-3'<br>Reverse 5'-ACCTCTGCCCAAGGCAGCAA-3'
Primers for RT-qPCR	
NPM1	Forward 5'-GGAGGTGGTAGCAAGGTTCC-3'<br>Reverse 5'-TTCACTGGCGCTTTTTCTTCA-3'
PD-L1	Forward 5'-TGGCATTTGCTGAACGCATTT-3'<br>Reverse 5'-TGCAGCCAGGTCTAATTGTTTT-3'
PARP1	Forward 5'-TGGAAAAGTCCCACACTGGTA-3'<br>Reverse 5'-AAGCTCAGAGAACCCATCCAC-3'
GAPDH	Forward 5'-ATCACCATCTTCCAGGAGCGA-3'<br>Reverse 5'-CCTTCTCCATGGTGGTGAAGAC-3'
Primers for ChIP-qPCR	
PD-L1 (-247~-24 bp)	Forward 5'-CTTCGAAACTCTTCCCGGTG-3'<br>Reverse 5'-ACCTCTGCCCAAGGCAGCAA-3'
PD-L1 (-469~-247 bp)	Forward 5'-AAACCAAAGCCATATGGGTC-3'<br>Reverse 5'-AGCCAACATCTGAACGCACC-3'
PD-L1 (-690~-469 bp)	Forward 5'-TAGAATAGGCTTCCGCAGCC-3'<br>Reverse 5'-CTAGAAAGTAGGTGTGTGTG-3'
Primers for plasmid construction	
3Flag-NPM1-WT	Forward 5‘-CGGGCTGCAGGAATTCATGGAAGATTCGATGGACATG-3‘<br>Reverse 5’-CCCCCTCGAGGTCGACTTAAAGAGACTTCCTCCACTGCCAGAGA-3’
3Flag-NPM1 △35	Forward 5’-CGGGCTGCAGGAATTCATGGAAGATTCGATGGACATG-3’<br>Reverse 5’-CCCCCTCGAGGTCGACTTAACCACCTTTTTCTATACTT-3’
3Myc-PARP1	Forward 5’-ATTTCTGAAGAAGACTTGACCGGTATGGCGGAGTCTTCGGATAAGCTCTA-3’<br>Reverse 5’-CCTCTAGATGCATGCTCGAGTTACCACAGGGAGGTCTTAAAATTGAAT-3’
sh-NPM1-1	Forward 5’-CCGGGGAATGTTATGATAGGACACTCGAGTGTCCTATCATAACATTCCTTTTTG-3’<br>Reverse 5’-AATTCAAAAAGGAATGTTATGATAGGACACTCGAGTGTCCTATCATAACATTCC-3’
sh-NPM1-2	Forward 5’-CCGGAGGTGGTAGCAAGGTTCCACTCGAGTGGAACCTTGCTACCACCTTTTTTG-3’<br>Reverse 5’-AATTCAAAAAAGGTGGTAGCAAGGTTCCACTCGAGTGGAACCTTGCTACCACCT-3’
sh-PARP1-1	Forward 5’-CCGGGAGTATGCCAAGTCCAACATTCTCGAGAATGTTGGACTTGGCATACTCTTTTTG-3’<br>Reverse 5’-AATTCAAAAAGAGTATGCCAAGTCCAACATTCTCGAGAATGTTGGACTTGGCATACTC-3’
sh-PARP1-2	Forward 5’-CCGGGGAACAAGGATGAAGTGAATTCTCGAGAATTCACTTCATCCTTGTTCCTTTTTG-3’<br>Reverse 5’-AATTCAAAAAGGAACAAGGATGAAGTGAATTCTCGAGAATTCACTTCATCCTTGTTCC-3’
pSIN-NPM1	Forward 5’-CCCGGACGAATTCTTCGAAATGGAAGATTCGATGGACATG-3’<br>Reverse 5’-TGCGGATCACTAGTGCTAGCTTAAAGAGACTTCCTCCACT-3’
pSIN-PARP1	Forward 5’-CCCGGACGAATTCTTCGAAATGGCGGAGTCTTCGGATAAGCTCTA-3’<br>Reverse 5’-TGCGGATCACTAGTGCTAGCTTACCACAGGGAGGTCTTAAAATTGAAT-3’
pSIN-PDL1 (human)	Forward 5’-CCCGGACGAATTCTTCGAAATGAGGATATTTGCTGTCTTTATAT-3’<br>Reverse 5’-TGCGGATCACTAGTGCTAGCTTACGTCTCCTCCAAATGTGTATCA-3’
pSIN-PDL1 (Mouse)	Forward 5’-CCCGGACGAATTCTTCGAAATGAGGATATTTGCTGGCATTATAT-3’<br>Reverse 5’-TGCGGATCACTAGTGCTAGCTTACGTCTCCTCGAATTGTGTATCAT-3’
sh-BRCA1	Forward 5’-CCGGCCTCACTTTAACTGACGCAATCTCGAGATTGCGTCAGTTAAAGTGAGGTTTTTG-3’<br>Reverse 5’-AATTCAAAAACCTCACTTTAACTGACGCAATCTCGAGATTGCGTCAGTTAAAGTGAGG-3’

Supplementary figure 1

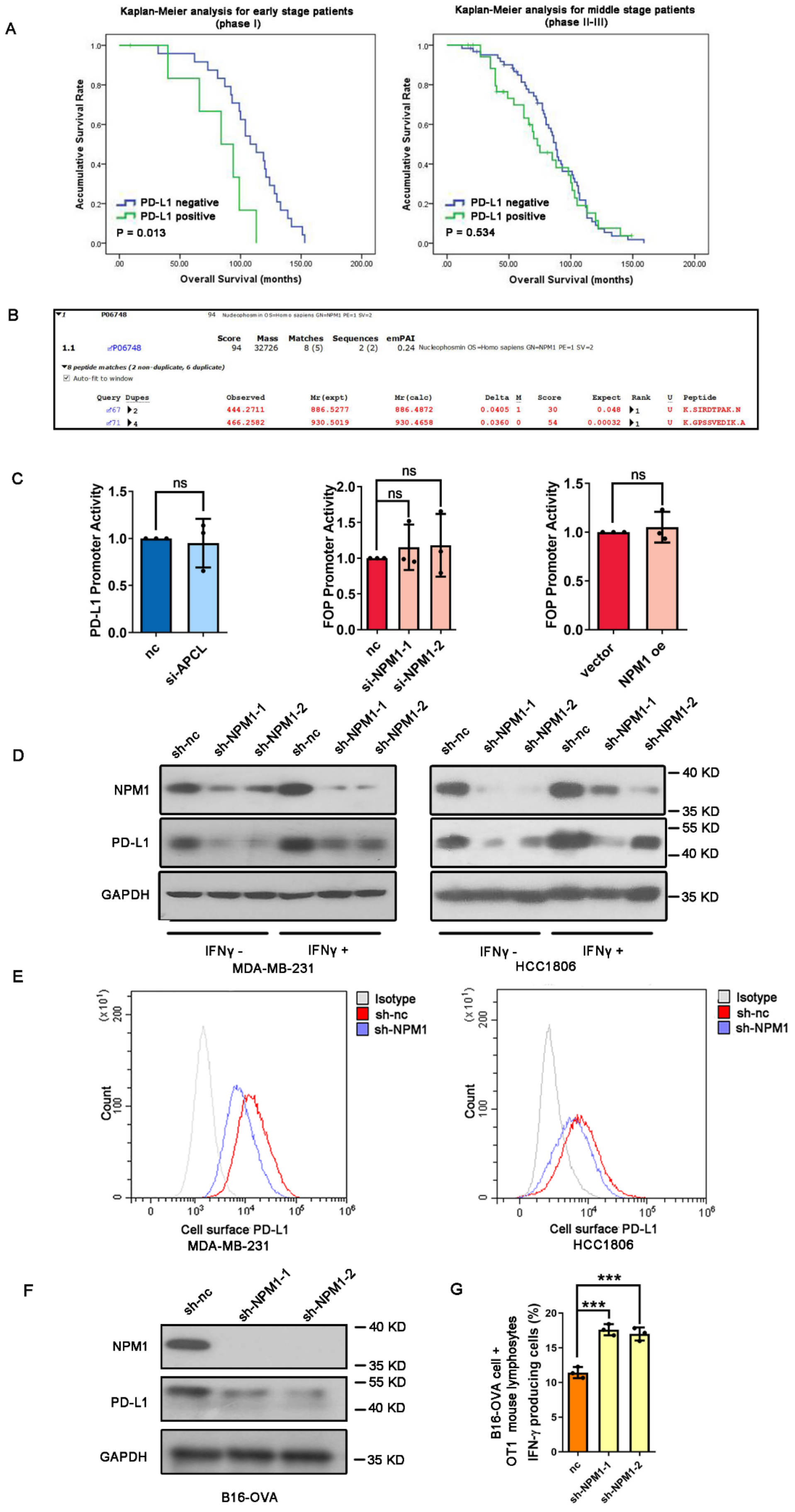

Supplementary Figure 1. (A) Kaplan Meier analysis for *PD-L1* in early stage (phase I, n=31) and middle stage (phase II~III, n=98) breast cancer patients. Data was analyzed by log-rank test. **(B)** Protein mass spectrometry data of NPM1. **(C)** Irrelevant si-RNA（si-APCL, left）and irrelevant promoter (FOP promoter, middle and right) were used as controls in *PD-L1* promoter activity assay. **(D)** NPM1 and PD-L1 protein expression in NPM1 stable knockdown cells (sh-NPM1-1, sh-NPM1-2) treated with (+) or without (-) IFN-γ was detected by Western blot. **(E)** Cell surface PD-L1 on MDA-MB-231 and HCC1806 cells was examined by flow cytometry. **(F)** NPM1 and PD-L1 protein expression in NPM1 stable knockdown B16-OVA cells. **(G)** OT-I mouse splenic lymphocytes were co-cultured with B16-OVA cells in a 6:1 ratio and IFN-γ positive lymphocytes were detected by flow cytometry. Data were presented as mean ± s.d. of three independent experiments. C left and right panel were analyzed by two-side Student's *t*-test. C middle panel and G were analyzed by one-way ANOVA+ two-side Dunnett test, ***$P < 0.001$. Source data are provided as a Source Data file.

Supplementary figure 2

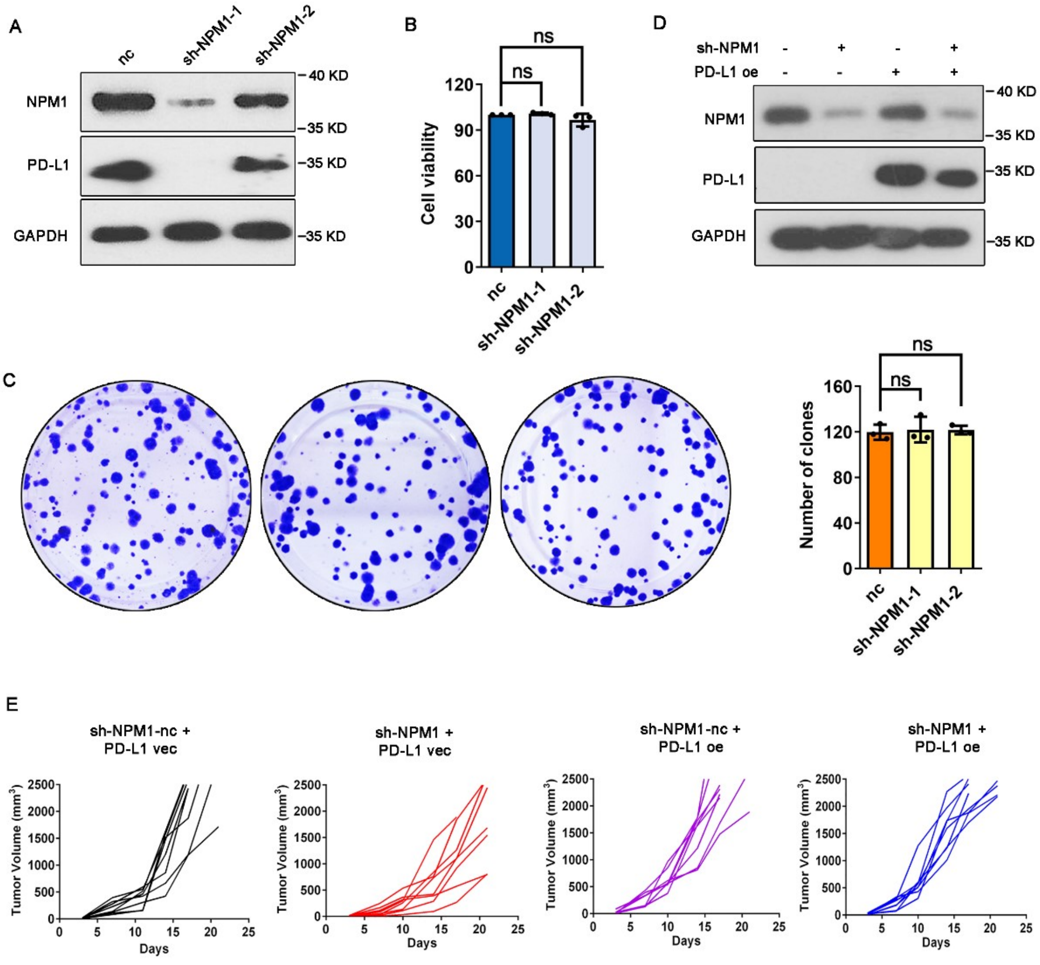

Supplementary Figure 2. (A) Protein expression of NPM1 and PD-L1 in 4T1 cells with NPM1 depletion (sh-NPM1-1, sh-NPM1-2) was analyzed by Western blot. **(B)** Viability of 4T1 cells with NPM1 knocked down was measured by MTS assay. **(C)** Anchorage-independent growth capacity of NPM1-depleted 4T1 cells was determined by clone formation assay. **(D)** Expression of NPM1 and PD-L1 in 4T1 cells with NPM1 knockdown or/and PD-L1 overexpression was detected by Western blot. **(E)** The tumor volume of every mouse in each group (n=8) was recorded twice a week for three weeks. Data were presented as mean ± s.d. of three independent experiments. B and C were analyzed by one-way ANOVA+ two- side Dunnett test; ns, not significantly different. Source data are provided as a Source Data file.

Spplementary figure 3

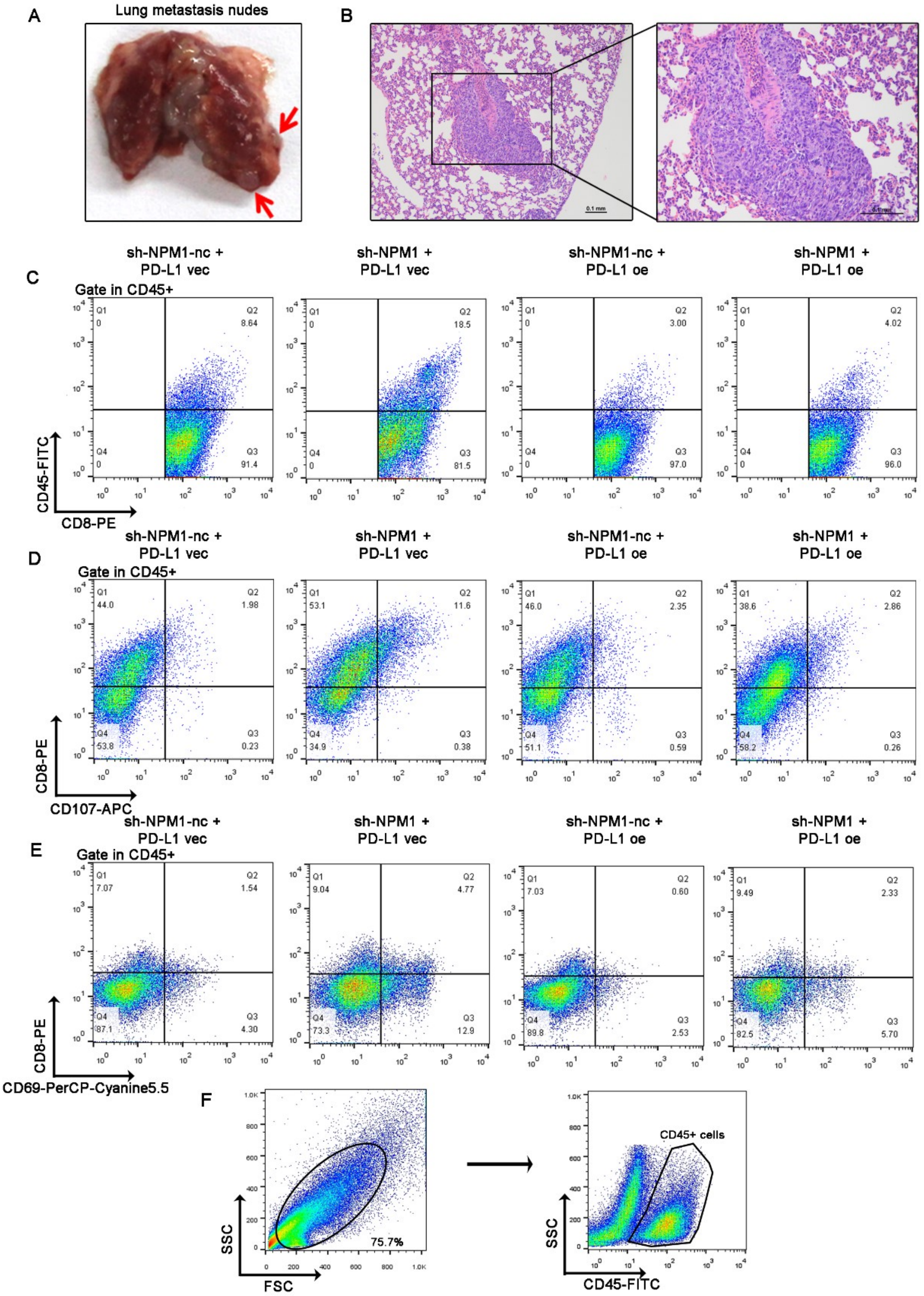

Supplementary Figure 3. (A) Representative image of metastatic nodules on the surface of lungs. **(B)** Representative haematoxylin and eosin (HE) staining of the metastatic lesions in the lungs. HE Staining was conducted in 32 animal samples. **(C-E)** CD45+, CD8+ (C) / CD45+, CD8+, CD107+ (D) / CD45+, CD8+, CD69+ (E) T cells in tumors from different groups were analyzed by flow cytometry. **(F)** Gating strategy for CD45+ cells.

Supplementary figure 4

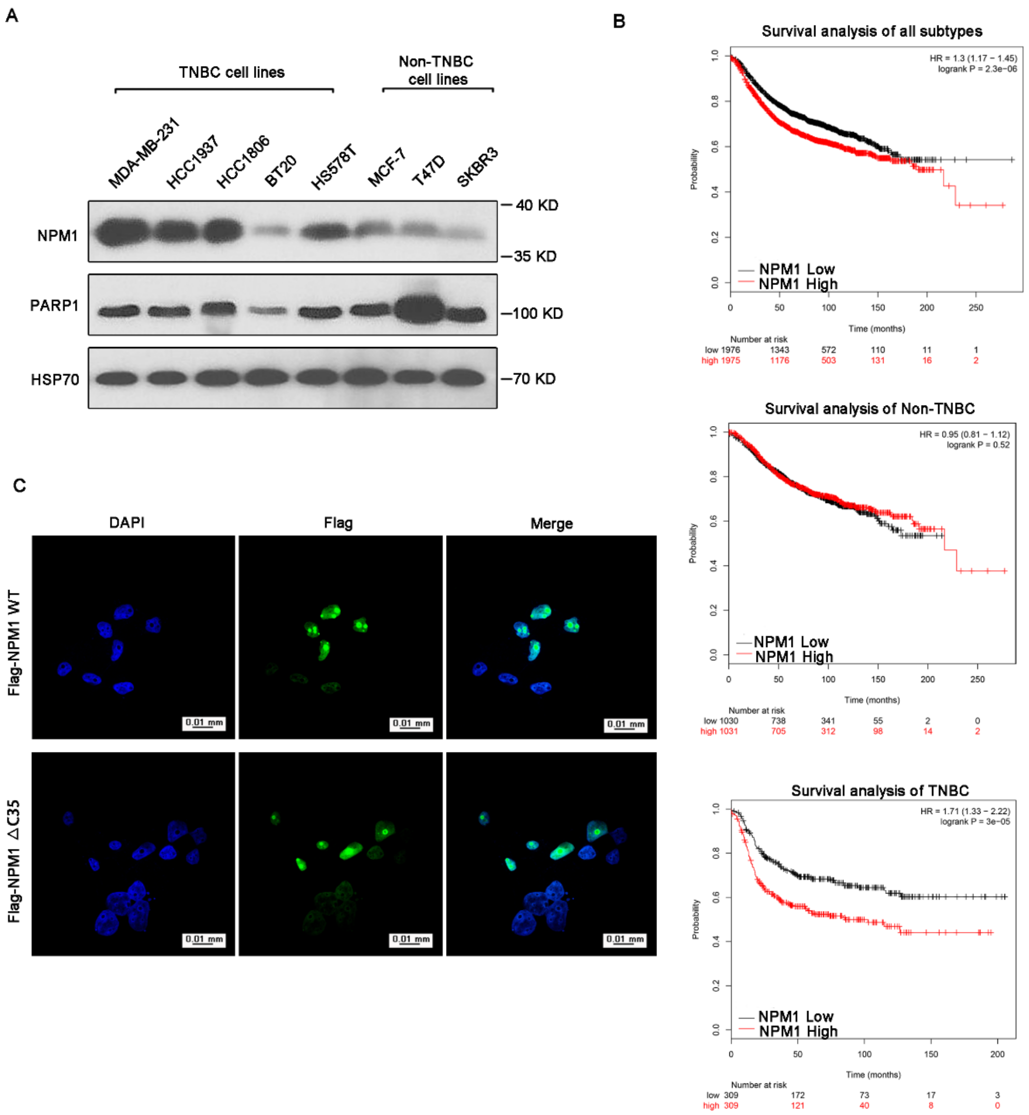

Supplementary Figure 4. (A) Protein expression of NPM1 and PD-L1 in TNBC and non-TNBC cell lines was detected by Western blot. **(B)** Survival analysis by Kaplan Meier Plotter based on NPM1 expression in breast cancer patients of all subtypes(top), non-TNBC (middle) and TNBC (bottom). Data was analyzed by log-rank test. **(C)** Flag-NPM1 WT and Flag-NPM1 ΔC35 plasmids were transfected into HEK293T cells, and IF assay was conducted to detect their cellular localization. Source data are provided as a Source Data file.

Supplementary figure 5

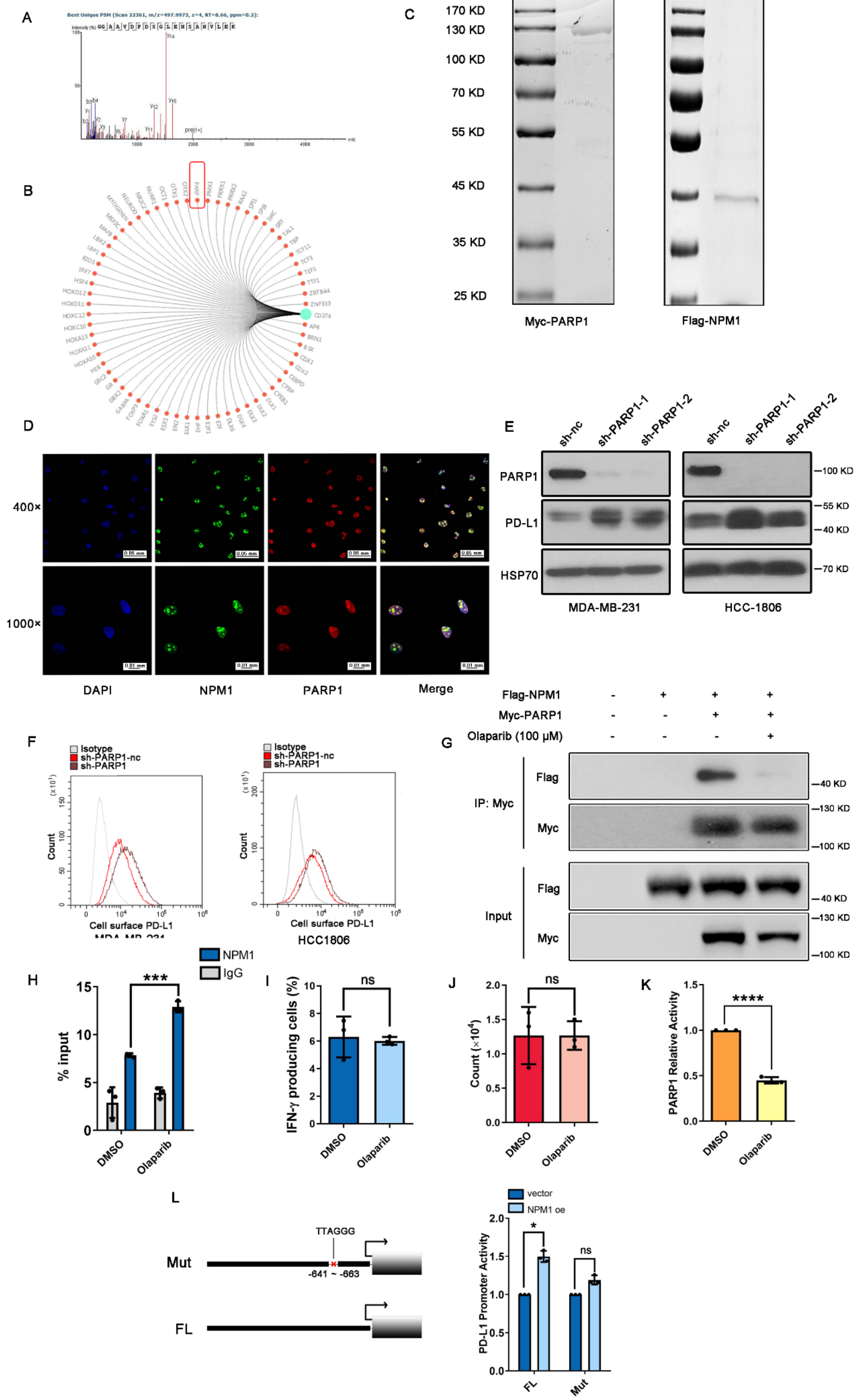

Supplementary Figure 5. (A) PARP1 was identified by mass spectrometry. **(B)** Predicted transcription regulators of *PD-L1* in GCBI database. **(C)** Myc-PARP1 (left) and Flag-NPM1 (right) proteins were purified and detected by Coomassie Brilliant Blue staining. **(D)** Localization of NPM1 and PARP1 in MDA-MB-231 cells was detected by IF. **(E)** PARP1 and PD-L1 protein expression in PARP1 stable knockdown cells (sh-PARP1-1, sh-PARP1-2) was detected by Western blot. **(F)** PD-L1 on the surface of MDA-MB-231 and HCC1806 cells with PARP1 stably knocked down was examined by flow cytometry. **(G)** HEK293T cells co-transfected with Flag-NPM1 and Myc-PARP1 plasmids were treated with or without olaparib. Cell lysates were immunoprecipitated with Myc antibody, and the precipitates were then detected with Flag antibody. **(H)** ChIP-qPCR of *PD-L1* promoter region was performed with NPM1 or control IgG antibody in MDA-MB-231 cells treated with 25 μM olaparib or DMSO for 48 h. **(I)** Human PBMCs were treated with 25 μM olaparib or DMSO for 24 h and IFN-γ producing was detected by flow cytometry. **(J)** The transwell assay for 25 μM olaparib or DMSO treated human PBMCs. **(K)** 4T1 cell was treated with 25 μM olaparib or DMSO for 48 h and the enzymatic activity of PARP1 was measured. **(L)** The structure of full length (FL) *PD-L1* promoter and TTAGGG deletion (Mut) PD-L1 promoter (left panel). *PD-L1* promoter activity in MDA-MB-231 cells transiently transfected with control plasmid for NPM1(vector), or NPM1 overexpression plasmid (NPM1 oe) was measured by dual-luciferase assay (right panel). Data were presented as mean ± s.d. of three independent experiments. H, I, J, K and L right panel were analyzed by two-side Student's *t*-test. *$P < 0.05$, ***$P < 0.001$, ****$P < 0.0001$; ns, not significantly different. Source data are provided as a Source Data file.

Supplementary figure 6

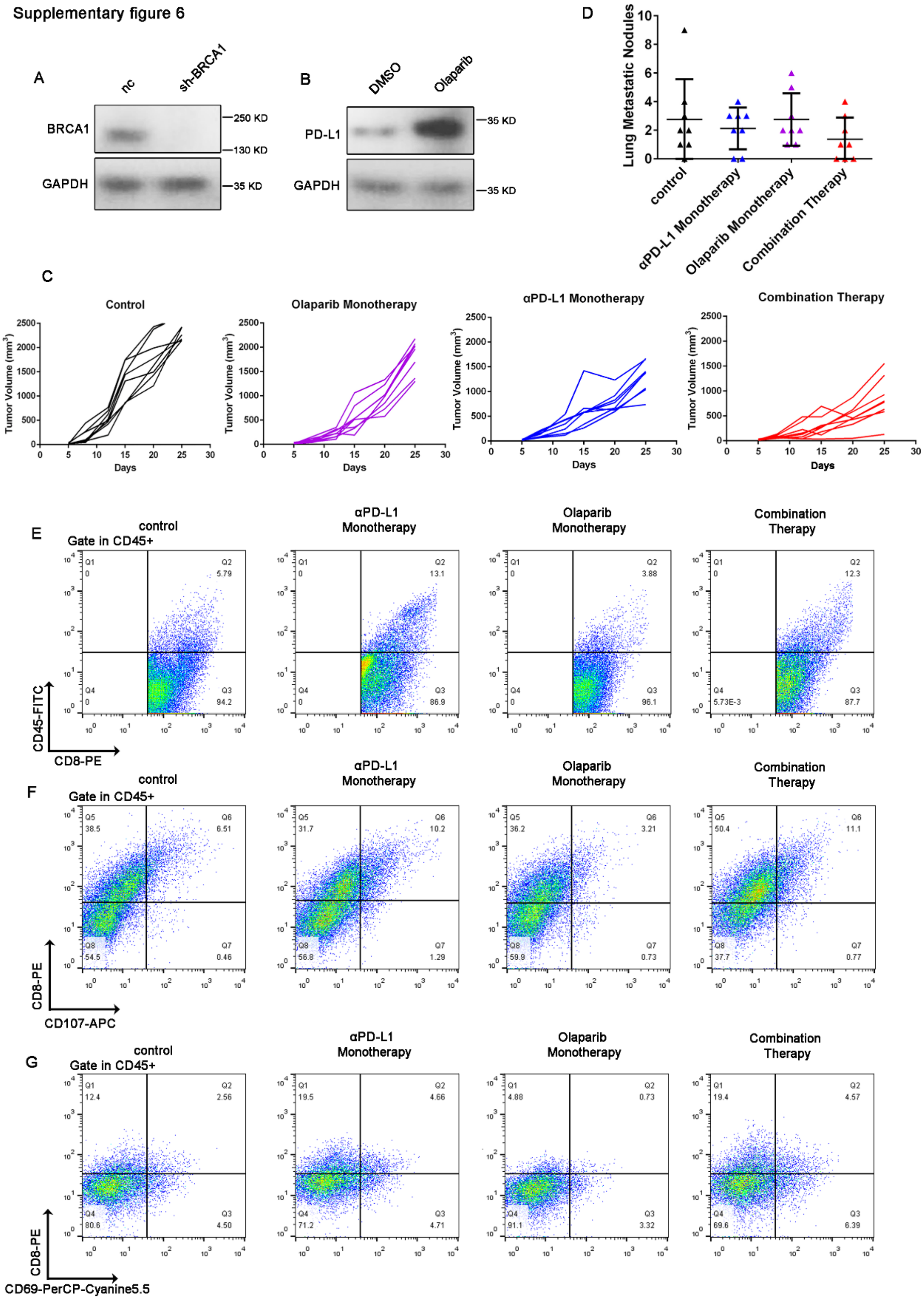

Supplementary Figure 6. (A) Western blot analysis of BRCA1 in 4T1 cells with BRCA1 knocked down. **(B)** Western blot analysis of PD-L1 in sh-BRCA1 4T1 cells treated with 25 μM olaparib. **(C)** The tumor volume of every mouse in each group (n=8) was recorded twice a week for three weeks. **(D)** The number of lung metastasis nodes of every mouse in each group was depicted, and the metastasis rate in teach group was indicated above the bar (n=8 animals). Data were presented as mean ± s.d. **(E-G)** CD45+, CD8+ (E) / CD45+, CD8+, CD107+ (F) / CD45+, CD8+, CD69+ (G) T cells in tumors from different treatment groups were analyzed by flow cytometry. Source data are provided as a Source Data file.

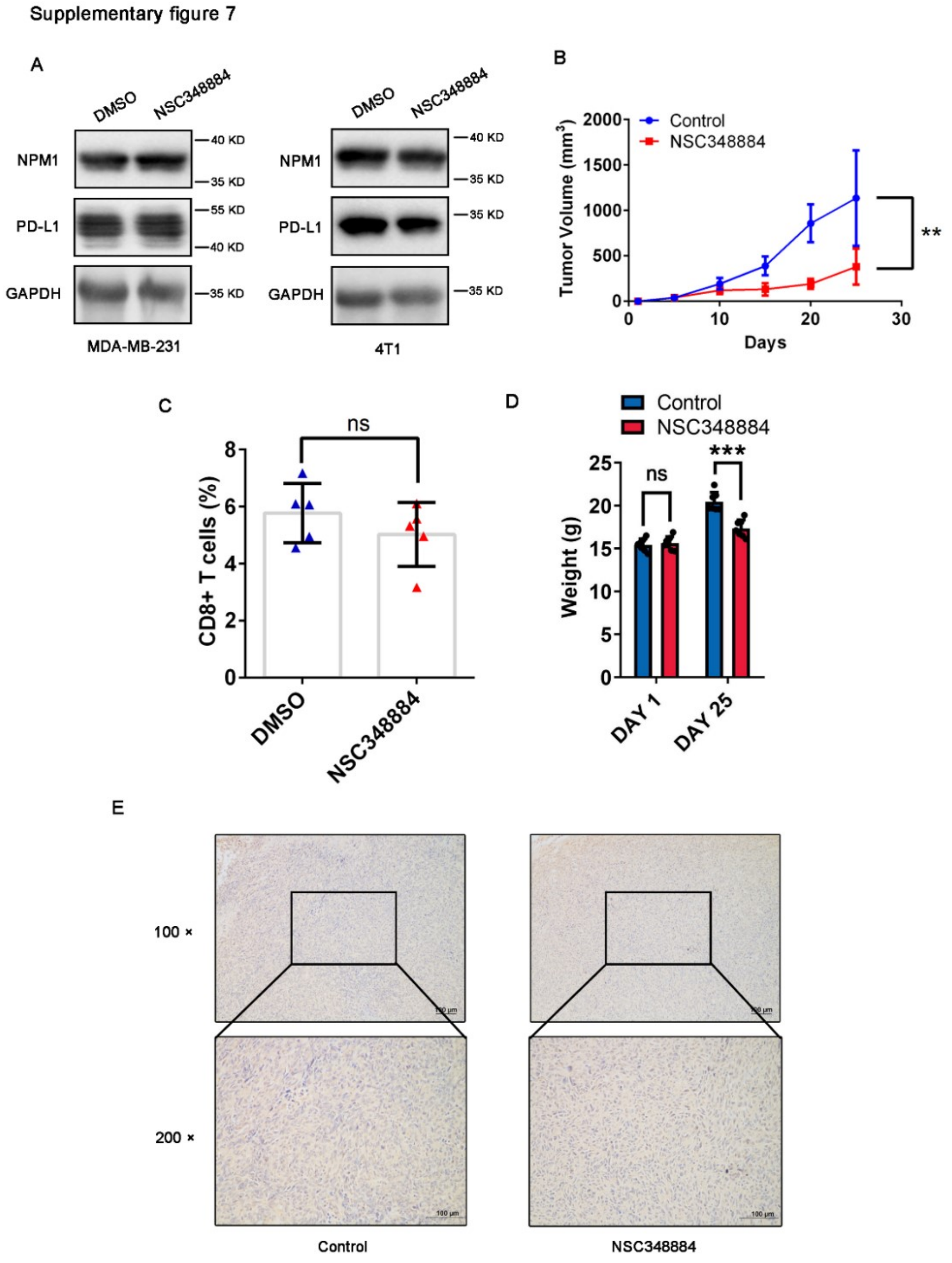

Supplementary Figure 7. (A) MDA-MB-231 and 4T1 cells were treated with DMSO or 4 μM NPM1 inhibitor NSC38884 for 48 h. The expression of NPM1 and PD-L1 were detected by Western blot. **(B)** The average tumor volume of control and NSC38884 treated group (n=7 animals). **(C)** CD45+CD8+ TILs were detected by flow cytometry (n=5 animals). **(D)** The average weight of control and NSC38884 treated group on day 1 and day 25 (n=7 animals). **(E)** PD-L1 expression was detected by IHC in 3 mouse tumor tissues of each group. Data were presented as mean ± s.d. B, C and D were analyzed by two-side Student's t-test. $^{**}P < 0.01$, $^{***}P < 0.001$; ns, not significantly different. . Source data are provided as a Source Data file.
