# Supplementary material for: NPM1 upregulates the transcription of PD-L1 and suppresses T cell activity in triple-negative breast cancer
Source: Nat Commun. 2020 Apr 3;11:1669. doi: 10.1038/s41467-020-15364-z (PMC7125142; doi:10.1038/s41467-020-15364-z)
Supplement: Supplementary file 3 — Description of Additional Supplementary Files [file 41467_2020_15364_MOESM3_ESM.pdf]

Description of Additional Supplementary Files

File Name: Supplementary Data 1
Description: Mass spectrometry data
