# Supplementary material for: NPM1 upregulates the transcription of PD-L1 and suppresses T cell activity in triple-negative breast cancer
Source: Nat Commun. 2020 Apr 3;11:1669. doi: 10.1038/s41467-020-15364-z (PMC7125142; doi:10.1038/s41467-020-15364-z)
Supplement: Supplementary file 5 — Reporting Summary [file 41467_2020_15364_MOESM5_ESM.pdf]

nature research

Corresponding author(s): Wuguo Deng

Reporting Summary

Nature Research wishes to improve the reproducibility of the work that we publish. This form provides structure for consistency and transparency in reporting. For further information on Nature Research policies, see Authors & Referees and the Editorial Policy Checklist.

Statistical parameters

When statistical analyses are reported, confirm that the following items are present in the relevant location (e.g. figure legend, table legend, main text, or Methods section).

n/a	Confirmed	
☐	☒	The exact sample size (*n*) for each experimental group/condition, given as a discrete number and unit of measurement
☐	☒	An indication of whether measurements were taken from distinct samples or whether the same sample was measured repeatedly
☐	☒	The statistical test(s) used AND whether they are one- or two-sided<br>*Only common tests should be described solely by name; describe more complex techniques in the Methods section.*
☒	☐	A description of all covariates tested
☒	☐	A description of any assumptions or corrections, such as tests of normality and adjustment for multiple comparisons
☐	☒	A full description of the statistics including central tendency (e.g. means) or other basic estimates (e.g. regression coefficient) AND variation (e.g. standard deviation) or associated estimates of uncertainty (e.g. confidence intervals)
☐	☒	For null hypothesis testing, the test statistic (e.g. *F*, *t*, *r*) with confidence intervals, effect sizes, degrees of freedom and *P* value noted<br>*Give P values as exact values whenever suitable.*
☒	☐	For Bayesian analysis, information on the choice of priors and Markov chain Monte Carlo settings
☒	☐	For hierarchical and complex designs, identification of the appropriate level for tests and full reporting of outcomes
☐	☒	Estimates of effect sizes (e.g. Cohen's *d*, Pearson's *r*), indicating how they were calculated
☐	☒	Clearly defined error bars<br>*State explicitly what error bars represent (e.g. SD, SE, CI)*

Our web collection on statistics for biologists may be useful.

Software and code

Policy information about availability of computer code

Data collection	FV10-ASW 1.7 Viewer, CytExpert(2.2.0.97)
Data analysis	GraphPad 6.0, SPSS Statistics 16.0, FlowJo V10, ELISACalc V 0.1

For manuscripts utilizing custom algorithms or software that are central to the research but not yet described in published literature, software must be made available to editors/reviewers upon request. We strongly encourage code deposition in a community repository (e.g. GitHub). See the Nature Research guidelines for submitting code & software for further information.

Data

Policy information about availability of data

All manuscripts must include a data availability statement. This statement should provide the following information, where applicable:

- Accession codes, unique identifiers, or web links for publicly available datasets
- A list of figures that have associated raw data
- A description of any restrictions on data availability

All data supporting the findings of this study are available with the article and Supplementary information or from the corresponding author upon reasonable request. The source data underlying Figures 1E,1F, 2B, 2E-M, 3A-C, 5B-I, 6A-G, 6I-M and 7B-C and Supplementary Figures 1C-D, 1F-G, 2A-E, 4A, 5E, 5G-L, 6A-D and 7A-D are provided as a Source Data file. Mass spectrometry data is provided in Supplementary data 1. PD-L1 promoter sequence was obtained from UCSC database

[https://genome.ucsc.edu]. We analyzed PD-L1 expression in breast cancer using TCGA data in Oncomine [http://www.oncomine.org]. Kaplan-Meier analysis of NPM1 in breast cancer was conducted in Kaplan-Meier Plotter [http://www.kmplot.com]. Transcription regulator prediction of PD-L1 was conducted in GCBI [https://www.gcbi.com.cn].

Field-specific reporting

Please select the best fit for your research. If you are not sure, read the appropriate sections before making your selection.

☒ Life sciences ☐ Behavioural & social sciences ☐ Ecological, evolutionary & environmental sciences

For a reference copy of the document with all sections, see nature.com/authors/policies/ReportingSummary-flat.pdf

Life sciences study design

All studies must disclose on these points even when the disclosure is negative.

Sample size	The sample size was determined according to previous literatures in the same research area for statistical power.
Data exclusions	Mice were excluded for poor body condition (e.g. after surgery).
Replication	All the experimental findings were reliably reproduced. The replication is indicated in corresponding figure legends and "Statistics and Reproducibility" part.
Randomization	The mice were weighed first and we found their weights had no significant difference. The mice were then numbered and grouped by Random Number method and were randomly and equally allocated into control and treated groups. Tumor specimens harvested from primary tumors of patients who underwent surgical resection at Sun Yat-sen University Cancer Center from 2000 to 2012. All samples were collected without bias. A set of tissue microarray including 140 breast cancer cases were bought from Outdo Biotech company (HBreD140Su04).
Blinding	Blinding was nor performed during animal treatment because of the differences in drug appearance. But in histopathological experiments the investigators were blinded.

Reporting for specific materials, systems and methods

Materials & experimental systems

n/a	Involved in the study
☒	☐ Unique biological materials
☐	☒ Antibodies
☐	☒ Eukaryotic cell lines
☒	☐ Palaeontology
☐	☒ Animals and other organisms
☐	☒ Human research participants

Methods

n/a	Involved in the study
☒	☐ ChIP-seq
☐	☒ Flow cytometry
☒	☐ MRI-based neuroimaging

Antibodies

Antibodies used	NPM1 (FC-61991, Invitrogen, 32-5200, TD268297), PD-L1 (Polyclonal, GeneTex, GTX104763, 42865), PD-L1 (Polyclonal, GeneTex, GTX31308, 821705316), PD-L1 (E1L3N(R), Cell Signaling Technology,13684, 7), PARP1 (46D11, Cell Signaling Technology,9532,5), Flag (D6W5B, Cell Signaling Technology,14793, 4), Myc (Proteintech, 16286-1-AP, 00053868), HSP70 (D1M6, Cell Signaling Technology, 46477,5), GAPDH (Polyclonal, Proteintech ,10494-1-AP, 00055858), CD274-PE (MIH1, eBioscience, 12-5983-42, 4338951) , Mouse IgG1 kappa Isotype Control-PE (P3.6.2.8.1, eBioscience; 12-4714-82), CD45-FITC (30-F11, eBioscience, 11-0451-82), CD8a-PE (53-6.7,12-0081-82; eBioscience, 4300679), CD107-APC(1D4B, eBioscience, MA5-28671), CD69-PerCP-Cyanine5.5 (H1.2F3, eBioscience, 45-0691-82), IFN-γ-APC(4S.B3, eBioscience, 17-7319-41, 4281150), CD8a-eFlour 615(53-6.7, eBioscience, 42-0081-82, 1931124), Granzyme B(Ploycloncal, abcam, ab4059, GR318-2367-5), Dylight 549 (polyclonal, A23320, Abbkine,) and Dylight 488 (polyclonal, A23210, Abbkine)
Validation	The information is described as: Antibody; Species reactivity; Validated Applications. 1. NPM1;Human, Mouse, Rat; ELISA, ICC, IF,IP, IHC, WB. 2. PD-L1(GTX104763); Human ; ICC, IF, IHC-Fr, IHC-P, WB. 3. PD-L1(GTX31308); Human, Mouse, Rat; ELISA, Flow, IHC, IHC-P, WB. 4. PD-L1(CST 13684); Human; WB, IP, IHC. Flow. 5.PARP1; Human, Mouse, Rat; WB, IP, IF. 6. Flag; WB, IP, IHC, IF, ChIP, Flow. 7. Myc; WB, IP, IF, ELISA. 8. HSP70; Human; WB, IHC. 9. GAPDH; Human, Mouse, Rat; WB, IP, IHC, IF, ELISA; 10. CD274-PE; Human; Flow, IF, FN, IHC; 11. CD45-FITC; Mouse; Flow, IF, IHC. 12. CD8a-PE; Mouse; Flow, IF, IHC, MISC, FN. 13.

CD107-APC; Mouse; Flow. 14. CD69-PerCP-Cyanine5.5; Mouse; Flow, IF. 15. IFN-γ-APC; Human; Flow. 16. CD8a-eFlour 615; Mouse; ICC, IF, IHC. 17. Granzyme B; Mouse, Rat, Human; ICC, IF, IHC, Flow.

Eukaryotic cell lines

Policy information about cell lines

Cell line source(s)	MDA-MB-231, BT-20, HS578T, SKBR3, HEK293T, B16 , HCC1806, HCC1937, MCF-7, T47D , 4T1 and Jurkat cells were obtained from American Type Culture Collection (ATCC, Manassas, VA, USA).
Authentication	None of the cell lines used were authentication.
Mycoplasma contamination	All cell lines tested negative for mycoplasma contamination.
Commonly misidentified lines (See ICLAC register)	No misidentified cell lines listed in ICLAC were used in this study.

Animals and other organisms

Policy information about studies involving animals; ARRIVE guidelines recommended for reporting animal research

Laboratory animals	Female Balb/C mice aged 5-6 weeks were quarantined for one week before use.
Wild animals	The study did not involve wild animals.
Field-collected samples	The study did not involve samples collected form the field.

Human research participants

Policy information about studies involving human research participants

Population characteristics	Please see the Supplementary Table 1 and 4.
Recruitment	Tumor specimens harvested from primary tumors of patients who underwent surgical resection at Sun Yat-sen University Cancer Center from 2000 to 2012. A set of tissue microarray including 140 breast cancer cases were bought from Outdo Biotech company (HBreD140Su04).

Flow Cytometry

Plots

Confirm that:

☒ The axis labels state the marker and fluorochrome used (e.g. CD4-FITC).

☒ The axis scales are clearly visible. Include numbers along axes only for bottom left plot of group (a 'group' is an analysis of identical markers).

☒ All plots are contour plots with outliers or pseudocolor plots.

☒ A numerical value for number of cells or percentage (with statistics) is provided.

Methodology

Sample preparation	For cell samples: The tumor cells were centrifuged(500rpm, 5min) and washed with cold PBS for 3 times. Cells were then resuspended in a final volume of 100 μL and treated with anti-PD-L1-PE antibody(1:20) for 20min. After PBS washing 3 times , cells were detected by FCM. Primary T cells were fixed and permeabilized with Intracellular Fixation & Permeabilization Buffer Set (88-8824-00; eBioscience) according to the manufacturer's instruction. Samples were then incubated with IFN-γ-APC antibody(1:20) for 30 min and analyzed by flow cytometry. For mice tumor tissues, the tumor tissues for flow cytometry were cut into small pieces and digested with 1 mg/ml collagenase type IV (C5138; Sigma) and 0.6 ku/ml DNAse (D5025; Sigma) for 2.5h. Samples were then filtrated to single-cell suspension. Cells were stained with CD45-FITC(1:20), CD8-PE(1:20) , CD107-APC(1:20), and CD69-PerCP-Cyanine5.5(1:20) . Subsequently, cells were analyzed by flow cytometry.
Instrument	CytoFLEX(Beckman Coulter), FACSCanto II(BD)
Software	CytExpert(2.2.0.97), FlowJo (v10)
Cell population abundance	Cell sorting not employed.
Gating strategy	Using the FSC/SSC gating, debris was removed by gating on the main cell population. Isotype control stained cells were used to distinguish between background staining and specific antibody staining.

☒ Tick this box to confirm that a figure exemplifying the gating strategy is provided in the Supplementary Information.
